# NF-**κ**B Signaling in the Brain of Autistic Subjects

**DOI:** 10.1155/2011/785265

**Published:** 2011-10-20

**Authors:** Mazhar Malik, Zujaja Tauqeer, Ashfaq M. Sheikh, Guang Wen, Amenah Nagori, Kun Yang, W. Ted Brown, Xiaohong Li

**Affiliations:** Department of Neurochemistry, New York State Institute for Basic Research in Developmental Disabilities, 1050 Forest Hill Road, Staten Island, New York, NY 10314, USA

## Abstract

Autism is a neurodevelopmental disorder characterized by problems in communication, social skills, and repetitive behavior. Recent studies suggest that apoptotic and inflammatory mechanisms may contribute to the pathogenesis of this disorder. Nuclear factor-*κ*B (NF-*κ*B) is an important gene transcriptional factor involved in the mediation of inflammation and apoptosis. This study examined the activities of the NF-*κ*B signaling pathway in the brain of autistic subjects and their age-matched controls. The NF-*κ*B activation is also determined in the brain of BTBR mice, which is a promising animal model for study of pathogenic mechanisms responsible for autism. Our results showed that the level of IKK*α* kinase, which phosphorylates the inhibitory subunit I*κ*B*α*, is significantly increased in the cerebellum of autistic subjects. However, the expression and phosphorylation of I*κ*B*α* are not altered. In addition, our results demonstrated that the expression of NF-*κ*B (p65), and the phosphorylation/activation of NF-*κ*B (p65) at Ser536 are not significantly changed in the cerebellum and cortex of both autistic subjects and BTBR mice. Our findings suggest that the NF-*κ*B signaling pathway is not disregulated in the brain of autistic subjects and thus may not be significantly involved in the processes of abnormal inflammatory responses suggested in autistic brain.

## 1. Introduction

Autism is a severe neurodevelopmental disorder of childhood characterized by impairments in social interaction, deficits in verbal and nonverbal communication, and restricted, repetitive, and stereotypical patterns of behavior and interests (DSMIV criteria, American Psychiatric Association, 1994). Many areas of the brain in autism show abnormalities that include decreased Purkinje cell counts in cerebellar hemispheres and vermis [1], loss of granular cells [2], and Purkinje cell atrophy [3, 4]. Susceptibility to autism is clearly attributable to genetic factors [5, 6], but the etiology of this disorder is unknown, and no biomarkers have yet been proven to be characteristic of autism. 

The BTBR T+*tf*J (BTBR) mice have been suggested to be a useful animal model for autism studies since they demonstrate low levels of sociability compared to the C57BL/6J (B6) mice [7–10]. The BTBR mice also exhibit an unusual pattern of ultrasonic vocalizations and high levels of self-grooming [9, 11–13] that may be homologous to the communication deficits and repetitive behaviors observed in autism. Thus, the BTBR strain of mice is currently a promising model for understanding the mechanisms that could be responsible for the pathogenesis of autism. 

Recently, emerging evidence points to central nervous system (CNS) inflammatory and apoptotic mechanisms being responsible for certain neuropsychiatric disorders including autism. Several inflammatory cytokines including tumor-necrosis-factor- (TNF-) *α*, interferon- (IFN-) *γ*, IL-1*β*, IL-6, and IL-8 are found to be elevated in the brain, serum, plasma, and cerebrospinal fluid (CSF) of autistic subjects [14–22]. In addition, previous studies, including ours, have found that the antiapoptotic Bcl2 protein is decreased, while the proapoptotic p53 protein is increased in the autistic brain [22, 23]. We also found that the BDNF-Akt-Bcl2 anti-apoptosis pathway is compromised in the frontal cortex of autistic subjects [22]. The BDNF-PI3K/Akt-Bcl2 signaling pathway plays an important role in inhibiting apoptosis and promoting neuronal survival. 

Nuclear factor-*κ*B (NF-*κ*B) is an important gene transcriptional factor that mediates cellular responses in inflammation, immunity, development, cell proliferation and apoptosis [24–30]. The inactive form of NF-*κ*B is localized to the cytoplasm and consists of DNA-binding p50 and p65 subunits and an inhibitory subunit, designated I*κ*B*α* [31]. Activation occurs via phosphorylation of I*κ*B*α* at Ser32 and Ser36, resulting in the ubiquitin-mediated proteasome-dependent degradation of I*κ*B*α* and the release and nuclear translocation of active NF-*κ*B dimmers [31]. The key regulatory step in this pathway involves activation of a high-molecular-weight IKappaB kinase (IKK) complex, consisting of three tightly associated IKK subunits. IKK*α* and IKK*β* serve as the catalytic subunits of the kinase. A large number of genes involved in cellular proliferation, apoptosis, and inflammation, are regulated upon activation of NF-*κ*B. These genes include antiapoptotic genes (Bcl2 and Bcl-xl), cell cycle-regulatory genes (cyclin D1), genes encoding adhesion molecules, chemokines, inflammatory cytokines, and genes involved in tumor metastases [26–28, 30, 32]. 

NF-*κ*B signaling pathway regulates gene transcription of Bcl2. NF-*κ*B is also an important transcriptional factor for cytokines. Activation of NF-*κ*B can be stimulated by TNF*α* [33]. TNF*α* induces NF-*κ*B p65 polyubiquitination and degradation, as well as termination of TNF*α*-mediated NF-*κ*B activation [34]. Although previous studies have shown that the BDNF-Akt-Bcl2 antiapoptotic pathway is downregulated and inflammatory cytokines are increased in autistic brain [22, 35], whether the NF-*κ*B signaling pathway is altered in the autistic brain has not yet been investigated. In the current study, we examined the entire NF-*κ*B signaling pathway in the cerebellum of 7 autistic subjects and their age-matched normal controls. We also determined the activity of NF-*κ*B in the frontal cortex of 6 autistic subjects and 6 BTBR mice in comparison with the controls. Our results show that the expression of IKK*α*, the kinase phosphorylating the inhibitory subunit I*κ*B*α*, is significantly increased in the cerebellum of autistic subjects as compared to age-matched controls. We failed to detect the IKK*β* expression in our samples by western blot Assay and reckon it is because the expression level of IKK*β* is very low. However, the expression and phosphorylation level of I*κ*B*α*, which is the downstream target of IKK*α* and IKK*β*, are not significantly changed in the autistic cerebellum. In addition, our results showed that the phosphorylation/activation of NF-*κ*B (p65) at Ser536 is not significantly altered in the cerebellum and frontal cortex of both autistic subjects and BTBR mice, as compared with the controls. These results suggest that NF-*κ*B signaling pathway is not deregulated in the brain of autistic subjects and BTBR mice.

## 2. Subjects and Methods

### 2.1. Study Subjects

Frozen human brain tissue of seven autistic subjects (mean age 8.1 ± 2.6 years) and seven age-matched normal subjects (mean age 8.4 ± 2.3 years) was obtained from the NICHD Brain and Tissue Bank for Developmental Disorders. Donors with autism fit the diagnostic criteria of the Diagnostic and Statistical Manual-IV, as confirmed by the Autism Diagnostic Interview-Revised. Participants were excluded from the study if they had a diagnosis of fragile X syndrome, epileptic seizures, obsessive-compulsive disorder, affective disorders, or any additional psychiatric or neurological diagnoses. The subjects' information is summarized in Table 1. 

Six female BTBR T+tfJ (BTBR) and six B6 mice were obtained from Jackson Laboratories (Bar Harbor, Me). Mice were housed for 24 hours with ad lib food and water to ease the stress before sacrifice. All procedures were conducted in compliance with the NIH Guidelines for the Care and Use of Laboratory Animals. 

### 2.2. Preparation of Brain Tissue Homogenate

Frozen cerebellum and frontal cortex tissues from human and mice were homogenized (10% W/V) in cold buffer containing 50 mM Tris-HCl (pH 7.4), 8.5% sucrose, 2 mM EDTA, 10 mM *β*-mercaptoethanol, and protease inhibitors cocktail (Sigma-Aldrich). The protein concentration was assayed by the BCA method [36, 37].

### 2.3. Western Blot Analysis

Antibodies IKK*α*, IKK*β*, IkB*α*, NF-*κ*B (p50/52), NF-*κ*B (p65), and phospho-NF-kB (p65) were obtained from commercial sources (Cell Signaling Technology, USA). For western blot analysis, brain homogenate samples in SDS sample buffer (20% glycerol, 100 mM Tris, pH 6.8, 0.05% w/v Bromophenol blue, 2.5% SDS (w/v), and 250 mM DTT) were denatured by heating at 100°C for three minutes. Forty to eighty micrograms of protein per lane per subject was loaded onto a 12% acryl-bisacrylamide gel and electrophoresed for 2 h at 120 V at room temperature (RT). The proteins were electroblotted onto a nitrocellulose membrane for 1 h at 100 V at 4°C. Protein blots were then blocked with 5% milk in phosphate buffered saline (PBS) with 1% Tween (PBST). After blocking, the blots were incubated with primary antibody overnight at 4°C (anti-IKK*α* 1 : 1000; anti-IKK*β* 1 : 500; anti-I*κ*B*α* 1 : 1000; anti-NF-*κ*B (p50/52) 1 : 500; anti-NF-*κ*B p65 1 : 500; anti-phospho-NF-*κ*B p65 1 : 500) followed by a secondary antibody incubation for 1 h at RT (goat anti-mouse IgG or goat anti-rabbit IgG, HRP conjugated, 1 : 5000, Sigma). After three washes in PBST (each time for 10 minutes), the blots were visualized using the ECL detection system (Amersham Pharmacia Biotech) and exposed to Hyper film ECL (Amersham Pharmacia Biotech). Sample densities were analyzed blind to the diagnosis, using Image J software. The densities of IKK*α*, I*κ*B*α*, and NF-*κ*B p65 expression bands, as well as the *β*-actin expression bands, were quantified with background subtraction. Statistical analyses were conducted using unpaired *t*-tests with significance established at *P* < 0.05. 

### 2.4. Immunohistochemistry

The immunohistochemistry studies were carried out on cerebellar cortex, which is the thin gray surface layer of the cerebellum, consisting of an outer molecular layer, a single layer of purkinje cells, and an inner granular layer. Six *μ*m paraffin sections from 10% formalin-fixed cerebellum specimens of autistic subjects and the age-matched controls were deparaffinized with Xylene (2X) and ethanol of 100% (2X), 80%, 50%, and 25% concentration and washed in TBS, 5 minutes each time. The sections were then incubated with I*κ*B*α* antibody (1 : 200) and NF-*κ*B p65 antibody (1 : 150) overnight in a moisture chamber at 4°C. After washing in 0.1 M PBS for 5 minutes, the sections were further incubated with secondary antibody (biotinylated horse anti-mouse IgG or biotinylated horse anti-rabbit IgG, VectaStain Elite ABC Kit, Vector Lab) for 30 minutes at RT, followed by incubation in Avidin-biotinylated peroxidase (VectaStain Elite ABC Kit) for 45 minutes at RT and in 0.0125 g DAB/25 mL 0.05 M TBS/1 drop 30% H_2_O_2_ for 10 minutes at RT. All sections were washed in sequence with TBS, 25%, 50%, 80%, and 100% ethanol (2X), and Xylene (2X) before mounting for view under the microscope (Zeiss, West Germany). 

### 2.5. Enzyme-Linked Immunosorbent Assay (ELISA)

A human phospho-NF-*κ*B p65 (Ser 536) Sandwich ELISA kit and a human phospho-I*κ*B*α* (Ser 32) Sandwich ELISA kit (Cell Signaling Technology, USA) were used, according to the protocol of the company, to measure the concentration of phospho-NF-*κ*B (p65) and phospho-I*κ*B*α* in the homogenates of cerebellum. 100 *μ*L of each standard and sample in duplicate added into appropriate wells in a 96-well microplate coated with anti-human phospho-NF-*κ*B (p65) and anti-human phospho-I*κ*B*α*, respectively, and incubated overnight at 4°C. The plates were washed 4 times with 1x Wash Solution (200 *μ*L each) and incubated with 100 *μ*L detection antibody for 1 hour at 37°C. Afterwards, the plates were washed 4 times with 1x Wash Solution (200 *μ*L each) and incubated with 100 *μ*L of HRP-linked secondary antibody for 30 minutes at 37°C followed by a further 5 times wash and incubation with 100 *μ*L of TMB Substrate for 10 minutes at 37°C in the dark. 100 *μ*L of Stop Solution was added to each well, and the plates were read within 30 minutes at 450 nm using Kinetic Microplate Reader (Molecular Devices). The average OD 450 of duplicate wells was plotted against the dilution factor for each test specimen on the same graph.

### 2.6. Statistical Analysis

We analyzed the statistical significance among groups with the unpaired *t*-test using the StatView 5.0 software (SAS Institute, Inc.). The samples shown in our study were all of the samples analyzed. All data is presented as means ± SE. Significance was accepted at *P* < 0.05 or better.

## 3. Results

### 3.1. IKK*α* Protein Expression Is Increased in the Cerebellum of Autistic Subjects

**Figure 1:**
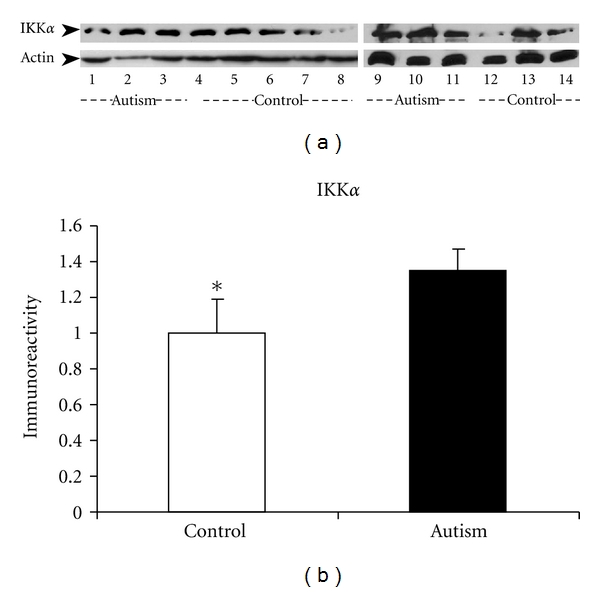
IKK*α* protein expression in the cerebellum of autistic subjects. (a) Two independent western blot studies on cerebellar homogenates using IKK*α* antibody (dilution 1 : 1000). Lanes 1–4 and 9–11 represent autistic subjects, and lanes 5–8 and 12–14 represent controls. The lower panel shows actin bands (MW: 48 kd), and the upper panel shows the IKK*α* bands (MW: 85 kd). (b) The blots shown in (a) were quantitated after being normalized by actin. Data are shown as mean ± SE. *P < 0.05 versus control group. White bars represent controls, and black bars represent autistic subjects.

western blot studies were conducted to examine IKK*α* expression in the cerebellum of autistic subjects and their age-matched controls. The results are shown in Figure 1. The bands representing the 85 kDa IKK*α* protein expression in the cerebellum were stronger in the autistic group than those in the control group (Figure 1(a)). Quantitative analysis showed that the mean value of IKK*α* expression was increased by 35% in the autistic subjects as compared with the control subjects (*P* < 0.05, Figure 1(b)). We failed to detect IKK*β* protein expression in the same set of samples, possibly due to very low expression in the cerebellum. 

### 3.2. The Protein Expression and Phosphorylation of I*κ*B*α* Inhibitory Subunit in the Cerebellum of Autistic Subjects

**Figure 2:**
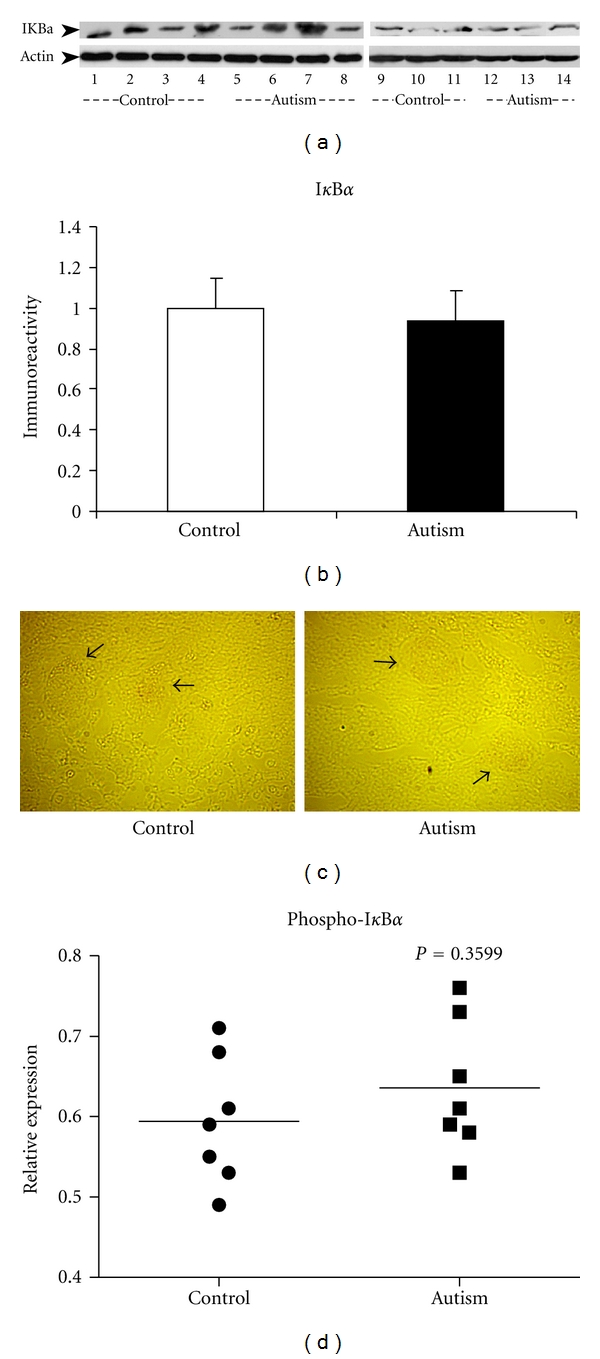
I*κ*B*α* inhibitory subunit expression in the cerebellum of autistic subjects. (a) Two independent western blot studies on cerebellar homogenates using I*κ*B*α* antibody (dilution 1 : 1000). Lanes 1–4 and 9–11 represent the controls, and lanes 5–8 and 12–14 represent the autistic subjects. The upper panel shows actin bands (MW: 48 kd), and the lower panel shows the I*κ*B*α* bands (MW: 35 kd). (b) Quantitative analysis after being normalized by actin. Data are shown as mean ± SE. White bars represent controls, and black bars represent autistic subjects. (c) Immunostaining of cerebellar sections using I*κ*B*α* antibody (dilution 1 : 200). The expression of I*κ*B*α* shown as very faint red color was detected in the neural cells (indicated by arrows) in autistic cerebellum (right panel), as well as in the matched controls (left panel). The image is viewed under high power microscopy. (d) Measurement of phospho-I*κ*B*α* in the cerebellum of 7 autistic subjects and the age-matched controls with ELISA. *P* = 0.36.

To examine the expression of the I*κ*B*α* protein and the downstream target of IKK*α* and IKK*β*, we carried out western blot studies on the cerebellum of autistic subjects and their age-matched controls. The results showed that there was no significant difference in I*κ*B*α* protein expression between the two groups (Figures 2(a) and 2(b)). To confirm this result, we examined I*κ*B*α* protein expression in both autistic cerebellum and the controls employing immunohistochemistry. Consistently, we detected no significant difference in I*κ*B*α* protein expression in the two groups (Figure 2(c)). To further determine the activities of I*κ*B*α*, we then examined the phosphorylation of I*κ*B*α* (on Ser 32) in the same set of samples using an ELISA approach and found that I*κ*B*α* phosphorylation was increased by 8.1%  ± 1.9% in the autistic cerebellum samples, but not significant as compared with the age-matched controls (*P* = 0.36, Figure 2(d)).

### 3.3. NF-*κ*B (p65) Protein Expression in the Cerebellum of Autistic Subjects

**Figure 3:**
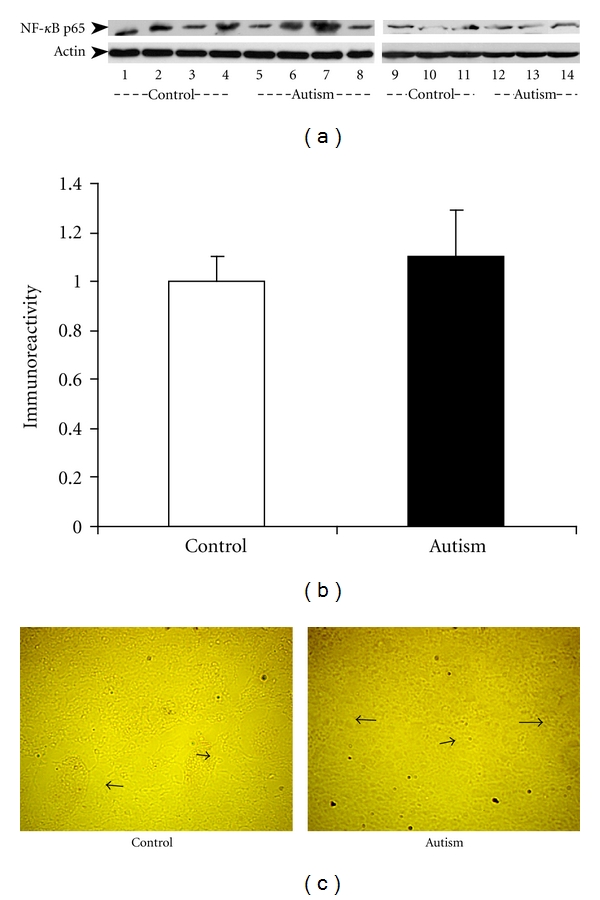
NF-*κ*B p65 protein expression in the cerebellum of autistic subjects. (a) Two independent western blot studies on cerebellar homogenates using NF-*κ*B p65 antibody (dilution 1 : 500). Lanes 1–4 and 9–11 represent the controls, and lanes 5–8 and 12–14 represent the autistic subjects. The lower panel shows actin bands (MW: 48 kd), and the upper panel shows the NF-*κ*B p65 protein bands (MW: 65 kd). (b) The blots shown in (a) were quantitated after being normalized by actin. Data are shown as mean ± SE. White bars represent controls, and black bars represent autistic subjects. (c) Immunostaining of cerebellar sections using NF-*κ*B p65 antibody (dilution 1 : 150). Very faint expression of NF-*κ*B p65 (red color) was detected in the neural cells (indicated by arrows) in autistic cerebellar (right panel), as well as in the matched controls (left panel). The image is viewed under high power microscopy.

The increased expression of IKK*α* kinase could result in an increased NF-*κ*B activity. To test this assumption, we examined the two subunits of NF-*κ*B (NF-*κ*B p65 and NF-*κ*B p50/52) in the cerebellum of the autistic subjects and their age-matched controls. We did not detect the expression of the NF-*κ*B p50/52 subunit and assume it is because the expression of NF-*κ*B p50/52 in the cerebellum is too low to be detected using western blot analysis. We successfully detected the expression of NF-*κ*B p65 (65 kDa) and found that the bands were slightly stronger in the autistic cerebellum than those in the control group (Figure 3(a)). However, quantitative analysis showed no significant difference in NF-*κ*B p65 expression between the autistic and the control subjects (*P* > 0.05, Figure 3(b)). This result was further confirmed by immunohistochemical studies using the NF-*κ*B p65 antibody. The positive immunoreaction was similar in both the autistic cerebellum and the controls (Figure 3(c)).

### 3.4. NF-*κ*B (p65) Activities in the Cerebellum and Frontal Cortex of Autistic Subjects

**Figure 4:**
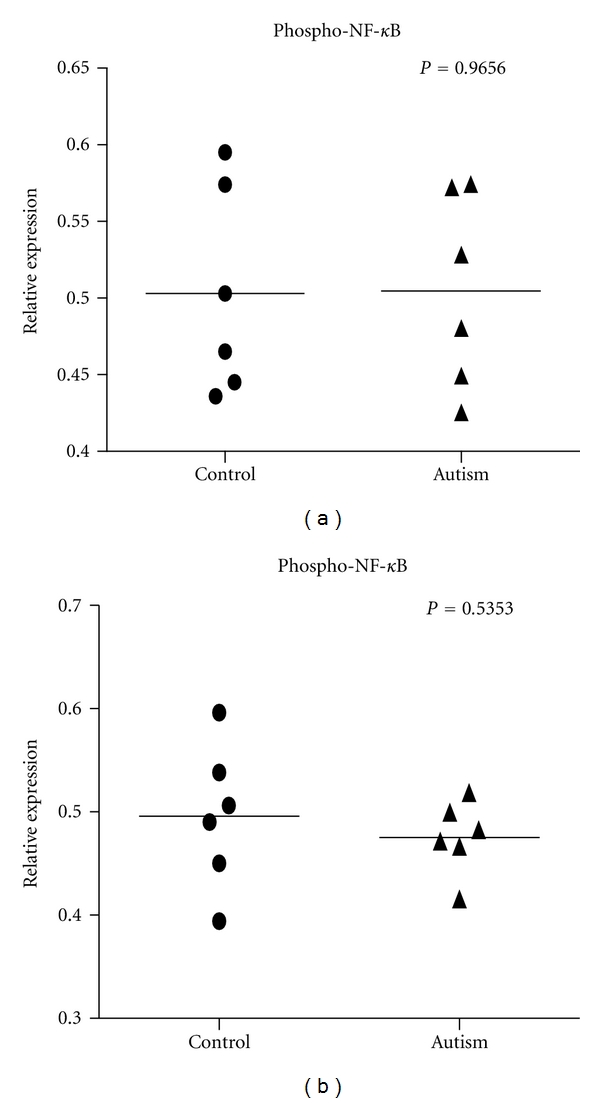
Phospho-NF-*κ*B p65 concentration in the cerebellum and cortex of autistic subjects. Measurement of phospho-NF-*κ*B p65 in the cerebellum ((a) *P* = 0.966) and cortex ((b) *P* = 0.535) of six autistic subjects and six age-matched controls with ELISA.

To further examine the activity levels of NF-*κ*B(p65) in the brain of autistic subjects, we examined NF-*κ*B(p65) phosphorylation (on Ser 536) in the cerebellum and frontal cortex of autistic subjects and the controls with an ELISA assay. Our results showed the mean values of NF-*κ*B(p65) phosphorylation were not significantly changed in the cerebellum (*P* = 0.966) and frontal cortex (*P* = 0.535) of autistic subjects, as compared to controls (Figures 4(a) and 4(b)). 

### 3.5. NF-*κ*B (p65) Activities in the Cerebellum and Frontal Cortex of BTBR Mice

**Figure 5:**
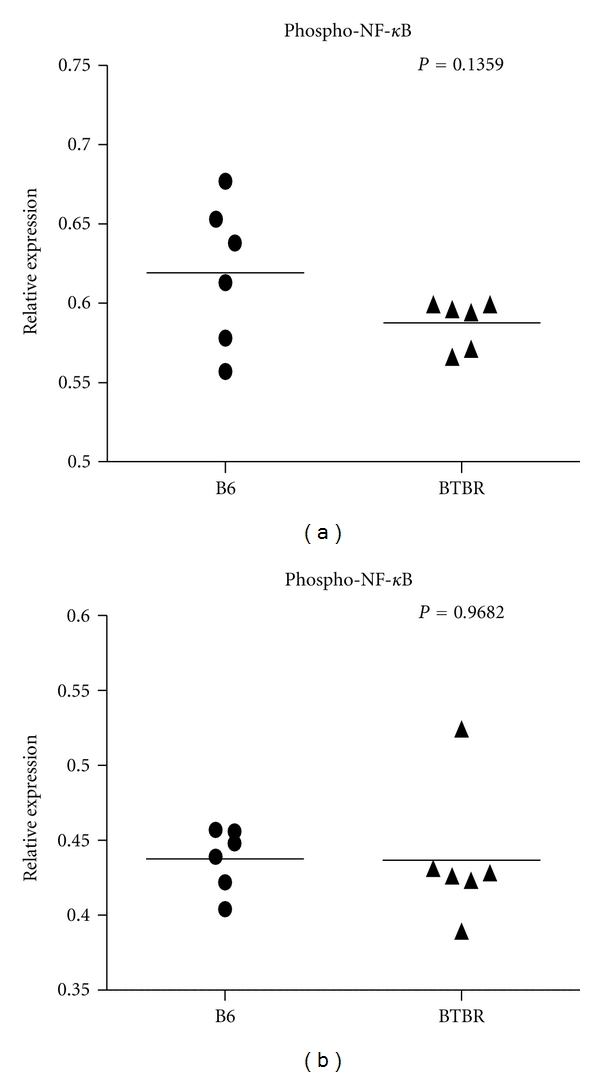
Phospho-NF-*κ*B p65 concentration in the cerebellum and cortex of BTBR mice. Measurement of phospho-NF-*κ*B p65 in the cerebellum ((a) *P* = 0.136) and cortex ((b) *P* = 0.968) of six BTBR mice and six age-matched control B6 mice with ELISA.

Since BTBR mice are currently a promising model for understanding the mechanisms that could be responsible for the pathogenesis of autism, we also examined the activity levels of NF-*κ*B(p65) in the cerebellum and frontal cortex of BTBR mice and B6 mice (control) by determining the NF-*κ*B(p65) phosphorylation on Ser 536 with an ELISA assay. Our results showed the mean values of NF-*κ*B (p65) phosphorylation were not significantly changed in the cerebellum (*P* = 0.136) and frontal cortex (*P* = 0.968) of BTBR mice, as compared to the control B6 mice (Figures 5(a) and 5(b)). 

## 4. Discussion

**Figure 6:**
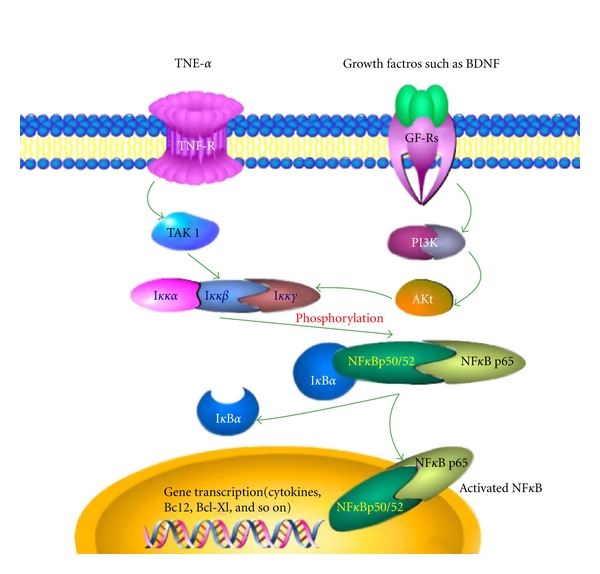
NF-*κ*B signal transduction pathway. NF-*κ*B heterodimer between p65 and p50/52 proteins is located in the cytosol complexed with the inhibitory protein I*κ*B*α*. Through the intermediacy of integral membrane receptors, a variety of extracellular signals (like TNF*α* or growth factor BDNF) can activate the enzyme I*κ*B kinase (IKK, consisting of Ikk*α*, *β*, and *γ*) via TAK1 or PI3K/Akt, respectively. IKK, in turn, phosphorylates the I*κ*B*α* protein, which results in dissociation of I*κ*B*α* from NF-*κ*B and eventual degradation by the proteosome. The activated NF-*κ*B is then translocated into the nucleus where it binds to specific sequences of DNA called response elements and initiates the gene transcription.

Emerging evidence suggests that apoptotic and inflammatory mechanisms may be related to the pathogenesis of autism. A number of studies have shown that apoptosis-related proteins (p53, Bcl2) and several inflammatory cytokines are altered in autistic brain [3, 4, 18–21]. NF-*κ*B is an important transcription factor that regulates cellular responses in inflammation and apoptosis. A large number of genes including the apoptosis-related Bcl2 and Bcl-xl, and inflammation-related cytokines, can be regulated upon activation of NF-*κ*B (see illustration of the NF-*κ*B signaling pathway in Figure 6). To further investigate the mechanisms that underlie the apoptotic and inflammatory changes in the autistic brain, we examined the activity of the transcriptional factor NF-*κ*B in the cerebellum and frontal cortex of autistic subjects and their age-matched controls. We also further determined NF-*κ*B activities in the cerebellum and frontal cortex of BTBR mice that model autism. This is the first study to investigate how the NF-*κ*B signaling pathway is regulated in the autistic brain. Our results demonstrated that the expression of IKK*α* was significantly increased in the autistic cerebellum as compared to the age-matched normal controls. IKK*α* is a kinase upstream of the NF-*κ*B signaling pathway and phosphorylates two serine residues located in the inhibitory subunit I*κ*B*α*. This phosphorylation event leads to I*κ*B*α* release from the NF-*κ*B complex and frees the NF-*κ*B complex to enter the nucleus and activate NF-*κ*B-dependent gene expression [33, 38]. The increased IKK*α* expression in the autistic brain implies a possible increased IKK*α* kinase activity and a possible increased phosphorylation of the inhibitory I*κ*B*α* subunit. However, by examining the expression of the I*κ*B*α* subunit, as well as the phosphorylation of I*κ*B*α* in the cerebellum of 7 autistic subjects and their age-matched controls, we did not detect significant differences in I*κ*B*α* expression and phosphorylation between the two groups. 

To further determine the activity levels of NF-*κ*B, we utilized western blot, immunohistochemistry, and ELISA approaches to examine the protein expression levels of both the NF-*κ*B p65 subunit and the NF-*κ*B p50/52 subunit, as well as the phosphorylation/activation levels of the two subunits in the brain of autistic subjects and their age-matched controls. Our results showed no significant difference in NF-*κ*B p65 expression between the autistic and the control subjects. The phosphorylation levels of NF-*κ*B p65 protein at Ser536 in the cerebellum and cortex of autistic subjects are also not significantly different from that of control subjects. The BTBR strain of mice is currently a promising model for understanding the mechanisms that could be responsible for the pathogenesis of autism since they demonstrate behaviors similar to autism. Thus, we also examined the phosphorylation levels of NF-*κ*B p65 protein at Ser536 in the cerebellum and cortex of BTBR mice. We found no significant differences in the phosphorylation levels of NF-*κ*B p65 protein at Ser536 in the cerebellum and cortex of BTBR mice as compared with control B6 mice. All these results point out that the activity of NF-*κ*B is not disregulated in the autistic brain. We could not detect protein expression of the NF-*κ*B p50/52 subunit in the cerebellum of autistic subjects or in control subjects. The very low expression level of this protein is the likely reason for its not being detectable with the western blot method. 

NF-*κ*B p65 phosphorylation at Ser 536 regulates activation, nuclear localization, protein-protein interactions, and transcriptional activities [39]. NF-*κ*B p65 is a subunit of the NF-*κ*B transcriptional factor that actively regulates expression of NF-*κ*B-dependent genes involved in apoptosis and inflammation. Antiapoptotic Bcl2 and Bcl-xl genes as well as genes for cytokines have been shown to be regulated through the activation of NF-*κ*B p65 [24, 40–42]. On one hand, constitutively expressed NF-*κ*B has been demonstrated to lead to resistance to cell death by different inducers of apoptosis and increased NF-*κ*B activity has recently been correlated with progression of different cancers, especially breast cancer, melanoma, and juvenile myelomonocytic leukemia [43]. On the other hand, studies have demonstrated that NF-*κ*B is negatively regulated by antiapoptotic Bcl2 protein and specifically activated in the course of apoptosis induced by serum withdrawal. This activation of NF-*κ*B was demonstrated to be necessary for the execution of the apoptotic program [44]. These findings suggest a complicated role of NF-*κ*B signaling in the regulation of apoptosis and inflammation.

Recently, a number of studies have suggested that apoptosis and inflammation in the central nerve system may be associated with autism [3, 4, 18–22]. We and others have found that the antiapoptotic factor Bcl2 is decreased in the autistic brain [3, 22, 23] and the BDNF-Akt-Bcl2 anti-apoptosis pathway is downregulated in the frontal cortex of autistic subjects [35]. In addition, a number of studies including ours found that the cytokines TNF*α*, IL-6, GM-CSF, IFN-*γ*, and IL-8 are significantly elevated in the brain and cerebrospinal fluid (CSF) of autistic subjects [18, 21, 22]. NF-*κ*B was shown to be the transcriptional factor that regulates the gene transcription of Bcl2 and inflammatory cytokines such as TNF*α* [24, 29, 42, 44]. Go et al. [45] have also reported that valproic acid inhibits neural progenitor cell death by activation of NF-*κ*B signaling pathway, which subsequently enhanced expression of antiapoptotic protein Bcl-XL. We expected the NF-*κ*B signaling activities altered in the autistic brain. However, our studies demonstrated that NF-*κ*B signaling is not significantly changed in autistic brain as compared with normal controls. These results could be explained as (1) the alteration of inflammatory cytokines and apoptosis-related proteins in the autistic brain may not be enough to stimulate or suppress the NF-*κ*B activities and (2) the consequence of dual role of NF-*κ*B in the response to inflammatory cytokines and apoptosis-related protein such as Bcl2. A number of studies have reported that NF-*κ*B can be activated in both transcriptionally activating and repressing forms [38, 46]. This dual role of NF-*κ*B has presented considerable complexities in understanding the mechanisms of NF-*κ*B action. 

## 5. Conclusions

Our studies have shown that although the expression of IKK*α* kinase, which is upstream of the NF-*κ*B signaling pathway, is significantly increased in the cerebellum of autistic subjects as compared with the age-matched controls, the phosphorylation/activation of the downstream I*κ*B*α* inhibitory subunit is not significantly enhanced in the cerebellum of autistic subjects. Our results further showed that both the expression and phosphorylation of NF-*κ*B p65 are not significantly altered in the cerebellum and cortex of autistic subjects as compared with the control subjects. The phosphorylation levels of NF-*κ*B p65 are also not significantly altered in the cerebellum and cortex of BTBR mice that model autism. These findings imply that NF-*κ*B signaling pathway is not disregulated in autism. 

## Figures and Tables

**Table 1:** Study subject information.

Case	Age	Sex	Group	PMI (h)	Seizure	Retardation	Medication	Cause of death
1	7	M	Control	12	−	−	Concerta, Clonidine	Drowning
2	8	M	Control	36	−	−	−	Drowning
3	4	F	Control	21	−	−	−	Lymphocytic myocarditis
4	9	F	Control	20	−	−	Albuterol, Zyrtec	Asthma
5	6	M	Control	18	−	−	−	Multiple injuries
6	14	M	Control	16	−	−	−	Cardiac Arrhythmia
7	11	F	Control	12	−	−	−	Asthma
8	7	M	Autism	20	−	−	−	Drowning
9	8	M	Autism	16	−	−	−	Drowning
10	4	F	Autism	13	−	−	−	Multiple injuries
11	9	F	Autism	24	−	−	−	Smoke inhalation
12	8	M	Autism	12	−	+	−	Drowning
13	14	M	Autism	12	+	+	−	Drowning
14	7	M	Autism	3	−	−	−	Cancer
